# Obstructive jaundice caused by a biliary mucinous cystadenoma in a woman: a case report

**DOI:** 10.1186/1752-1947-7-278

**Published:** 2013-12-30

**Authors:** Pramodh C Chandrasinghe, Chandika Liyanage, Kemal Ismail Deen, Suraj Ruwan Wijesuriya

**Affiliations:** 1 Department of Surgery, University Surgical Unit, North Colombo Teaching Hospital, Ragama, Sri Lanka

**Keywords:** Benign liver cyst, Liver cyst, Mucinous cystadenoma, Obstructive jaundice

## Abstract

**Introduction:**

Mucinous cystadenoma of the liver is a rare (less than 5%) neoplasm. This condition is more common in young women and accounts for non-specific symptoms. Cyst adenomas commonly affect the intrahepatic system (90%) and are rarely found in the extrahepatic biliary system or affecting both the systems.

**Case presentation:**

A 39-year-old Sinhalese woman presented with features of obstructive jaundice and was found to have a biliary neoplasm on imaging. In the absence of a definitive diagnosis despite extensive imaging she underwent preoperative endoscopic biliary drainage followed by a left hemihepatectomy with Roux-en-Y hepaticojejunostomy. A pathological examination of the specimen revealed an obstruction of the bile duct caused by a biliary mucinous cystadenoma affecting both the intrahepatic and extrahepatic systems.

**Conclusions:**

Biliary mucinous cystadenoma rarely present with obstructive jaundice affecting both intrahepatic and extrahepatic ducts. Exhaustive investigation might not help in the diagnosis and may need to be treated based on clinical judgment. The definitive treatment modality is surgery due to its malignant potential. The operative procedure is technically demanding and is best performed at specialist centers to minimize complications.

## Introduction

Cystadenomas arising from the biliary epithelium account for less than 5% of the cystic neoplasms of the liver [1]. Clinical presentation of this condition varies significantly and obstructive jaundice is a rare presentation [2]. We report the case of a woman presenting with obstructive jaundice caused by an intrahepatic biliary cystadenoma.

## Case presentation

**Figure 1:**
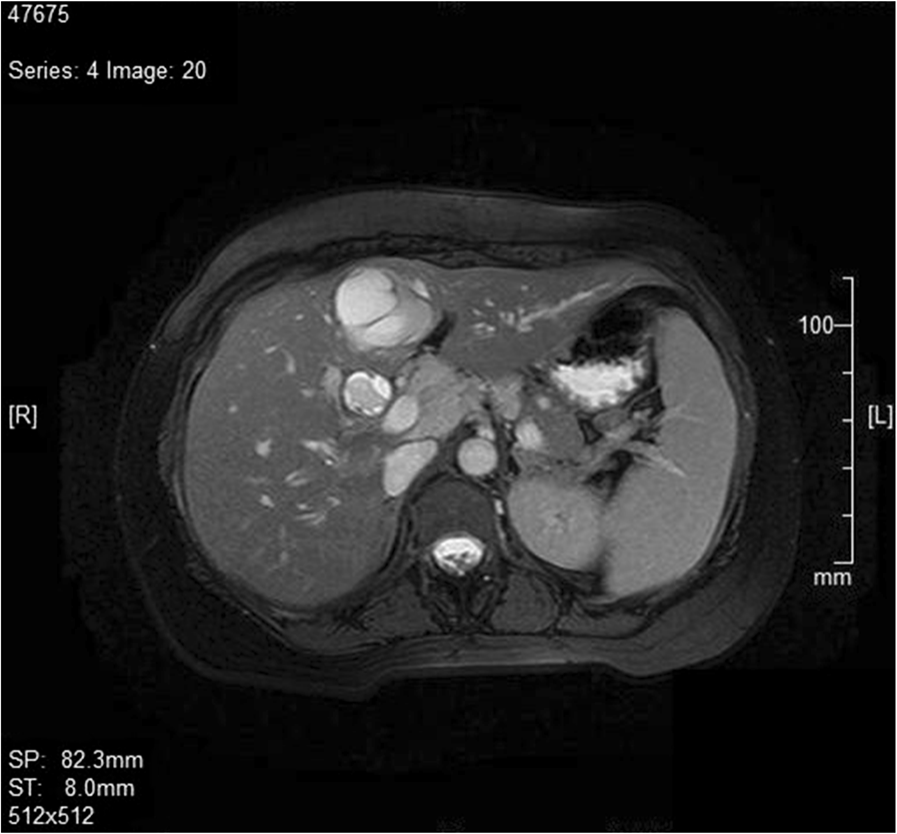
Magnetic resonance imaging image of the liver showing the dilated common bile duct with a filling defect within it indicating the tumor extending.

**Figure 2:**
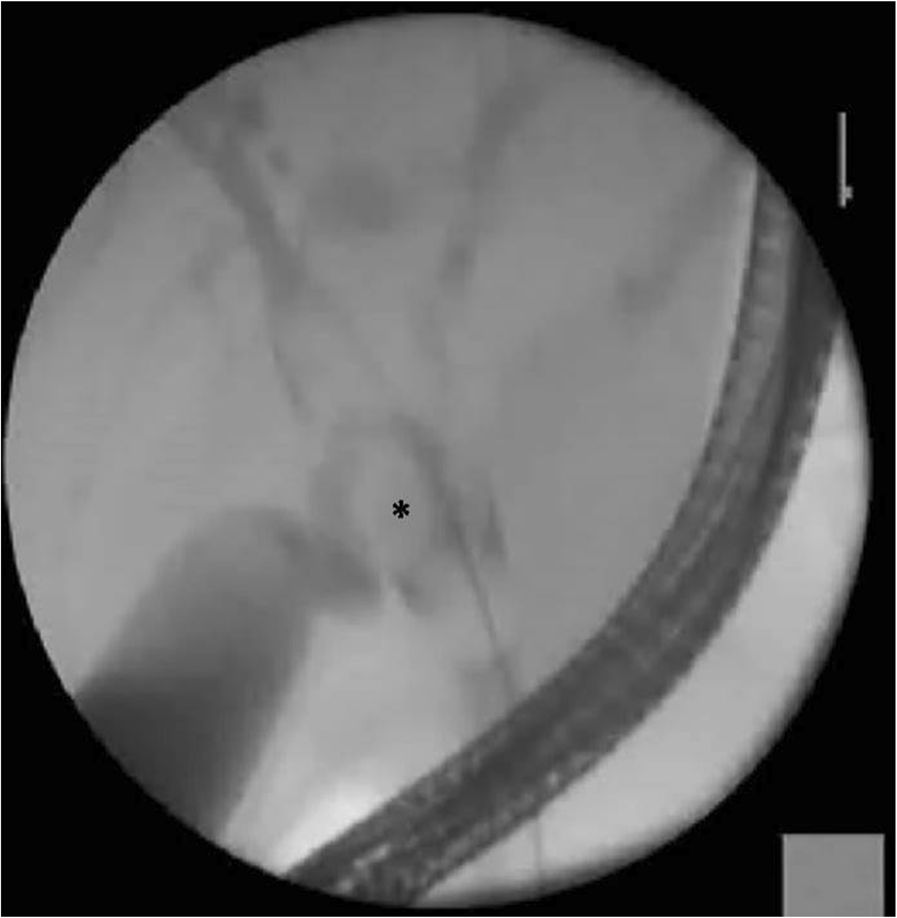
Endoscopic retrograde cholangiopancreatogram image of the cystadenoma seen as a filling defect at the hilum (marked with an *).

A 39-year-old Sinhalese woman presented with obstructive jaundice of 3 months’ duration. She had deeply icteric sclera with evidence of pruritus. An abdominal examination revealed mild hepatomegaly. Serum bilirubin, alkaline phosphatase and gamma-glutamyl transferase levels were significantly elevated while the transaminases were moderately high. An ultrasound examination was suggestive of a cystic lesion in segment IV of her liver with dilated intrahepatic ducts. A triphasic computed tomography (CT) scan revealed a non-enhancing cystic lesion on segment IV with intrahepatic duct dilatation. A subsequent magnetic resonance imaging (MRI) scan of her liver and magnetic resonance cholangiopancreatogram (MRCP) confirmed the cystic lesion in her liver and a mass lesion occupying the common hepatic duct and proximal common bile duct up to the level of her duodenum (Figure 1). Her serum carbohydrate antigen (CA) 19–9 was over 1000U/mL (normal <40U/mL). In the interim period she underwent an endoscopic retrograde cholangiopancreatogram (ERCP) and temporary stent placement to relieve biliary obstruction (Figure 2). Brush cytology obtained during ERCP did not reveal abnormal cells. We could not arrive at a diagnosis with the available evidence. As definitive treatment she underwent left hemihepatectomy and excision of extrahepatic bile duct and reconstruction by hepaticojejunostomy.

**Figure 3:**
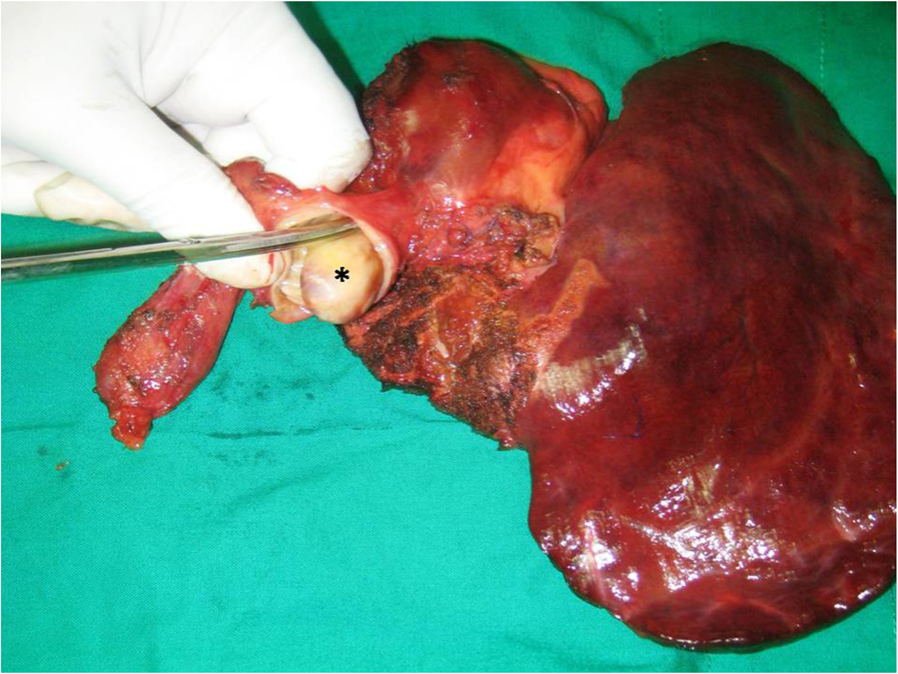
**Extended left hemihepatectomy specimen with the tumor within the common bile duct.** The ‘*’ indicates the mucinous cystadenoma within the dilated common bile duct.

Examination of the specimen revealed a cystic lesion located in segment IV of her liver with extension of a solid mass along the duct of segment IV, up to her common bile duct completely obstructing the extrahepatic biliary system (Figure 3). A histological examination of multiple cross-sections of the specimen revealed a cystic space lined by a simple mucin-secreting columnar to low-cuboidal epithelium. The subepithelial tissue resembled ovarian stromal tissue. None of the examined sections revealed papillomatosis or nuclear atypia. No foci of malignant transformation were observed.

She had an uneventful recovery. She was symptom free at her 3-month postoperative follow-up visit with no clinical or ultrasonic evidence of recurrence.

## Discussion

Biliary cystadenomas are rare benign tumors with a potential for malignant change [3]. Of the two subtypes the mucinous type accounts for 95% of cases whereas the rest are of the serous type [4]. Mucinous cystadenomas typically occur in middle-aged women. Although the etiology is uncertain, it is thought to occur either as a result of the development of ectopic foci of primitive foregut sequestered within the liver or due to the obstruction of the congenitally aberrant bile duct [5]. Most patients with a biliary cyst adenoma present with nonspecific symptoms such as epigastric pain or a palpable epigastric mass. Obstructive jaundice due to common bile duct obstruction is a rare occurrence [2,6].

Tumor markers are nonspecific, although CA 19–9 levels are known to be high. Intracystic fluid CA 19–9 levels are particularly elevated in cystadenomas. This patient had a serum CA 19–9 level of over 1000U/mL (normal <40U/mL).

An ultrasound scan and triphasic CT scan of the abdomen are considered adequate to arrive at the diagnosis in most cases. MRI and MRCP were performed in this patient because of atypical presentation with obstructive jaundice. On ultrasound scanning, the hepatic cystadenomas appear as anechoic lesions with septations. On a CT scan, the tumor appears as low-density areas with focal enhancement after contrast administration. The septa and the mural nodules may be visible. However, these imaging modalities may not distinguish benign lesions from malignant type but malignant transformation can be suspected by thickened cyst wall, calcification, and papillary projections within the separations [7]. Kim *et al*. studying radiological features of 25 cysts on CT images concluded that upstream biliary dilatation, transient hepatic attenuation difference, location in the left lobe and the presence of fewer than three cysts to be in favor of a neoplasm rather than a simple cyst [8]. This patient had a single cyst located on her left lobe with biliary duct dilatation which confirmed a neoplasm. Absence of clear radiological features to differentiate the benign from malignant neoplasms makes it necessary to undertake surgical resection.

The histopathological evaluation of the cyst wall is of importance to differentiate the mucinous cystadenoma from other types of liver cysts and to identify malignant transformation. Other cyst types needing differentiation are the simple cyst, cystic hamartomas and the recently described biliary intraductal papillary neoplasm (IPN). Particular characteristics of the mucinous cystadenoma are the single cuboidal epithelium lining and ovarian-type subepithelial stroma [9]. Both the above characteristic features were observed in the surgical specimen in this patient. An IPN on histology would show papillomatosis on the surface lining whereas a simple cyst is devoid of subepithelial ovarian stroma which differentiate these two from a cystadenoma. Immunohistochemical markers have also been studied in small cohorts of patients and Zen *et al*. [10] have observed cytokeratin 7 to have a higher positive predictive value for benign cystic mucinous neoplasm than papillary mucinous neoplasms. However, there are no definitive tumor markers found with high diagnostic accuracy.

Radical surgical excision of the tumor with a 2cm margin is the accepted treatment of choice for mucinous cyst adenoma [3,11]. Lesser procedures have an unacceptable recurrence rate. This patient underwent left hemihepatectomy with complete excision of her bile duct and hepaticojejunostomy to her right hepatic duct. Recurrence rate after radical resection is reported to be 10% [9]. Postoperative follow up of the patients is warranted to detect postoperative complications in the biliary anastomosis and recurrence of the tumor.

## Conclusions

Biliary cystadenomas rarely present with features of obstructive jaundice and pose a diagnostic challenge. Availability of imaging modalities may not help to distinguish these lesions from cholangiocarcinomas. They are best treated radically as they have a definite potential to transform into malignant tumors. Preoperative endoscopic biliary drainage was safely performed in this situation but more evidence is needed before it is incorporated into routine practice, hence a selective approach is suitable. Surgical resection is technically demanding and is best performed at specialized hepatobiliary centers.

## Consent

Written informed consent was obtained from the patient for publication of this case report and accompanying images. A copy of the written consent is available for review by the Editor-in-Chief of this journal.

## Abbreviations

CA: Carbohydrate antigen; CT: Computed tomography; ERCP: Endoscopic retrograde cholangiopancreatogram; IPN: Intraductal papillary neoplasm; MRCP: Magnetic resonance cholangiopancreatogram; MRI: Magnetic resonance imaging.

## Competing interests

The authors declare that they have no competing interests.

## Authors’ contributions

PCC was involved in acquiring patient data, literature survey on the subject, patient management and preparing the manuscript. CL and SRW were responsible for carrying out the investigative procedures and surgery on the patient. KID and SRW were also actively involved in preparing the manuscript and critical appraisal. All authors read and approved the final manuscript.
